# Screening of DNA Damage Repair Genes Involved in the Prognosis of Triple-Negative Breast Cancer Patients Based on Bioinformatics

**DOI:** 10.3389/fgene.2021.721873

**Published:** 2021-08-02

**Authors:** Nan Wang, Yuanting Gu, Jiangrui Chi, Xinwei Liu, Youyi Xiong, Chaochao Zhong, Fang Wang, Xinxing Wang, Lin Li

**Affiliations:** ^1^Department of Breast Surgery, The First Affiliated Hospital of Zhengzhou University, Zhengzhou, China; ^2^Department of Plastic Surgery, The First Affiliated Hospital of Zhengzhou University, Zhengzhou, China

**Keywords:** DNA damage response, prognostic model, bioinformatics and computational biology, triple-negative breast cancer, biomarker discovery

## Abstract

**Background:** Triple-negative breast cancer (TNBC) is a special subtype of breast cancer with poor prognosis. DNA damage response (DDR) is one of the hallmarks of this cancer. However, the association of DDR genes with the prognosis of TNBC is still unclear.

**Methods:** We identified differentially expressed genes (DEGs) between normal and TNBC samples from The Cancer Genome Atlas (TCGA). DDR genes were obtained from the Molecular Signatures Database through six DDR gene sets. After the expression of six differential genes were verified by quantitative real-time polymerase chain reaction (qRT-PCR), we then overlapped the DEGs with DDR genes. Based on univariate and LASSO Cox regression analyses, a prognostic model was constructed to predict overall survival (OS). Kaplan–Meier analysis and receiver operating characteristic curve were used to assess the performance of the prognostic model. Cox regression analysis was applied to identify independent prognostic factors in TNBC. The Human Protein Atlas was used to study the immunohistochemical data of six DEGs. The prognostic model was validated using an independent dataset. Gene Ontology and the Kyoto Encyclopedia of Genes and Genomes analysis were performed by using gene set enrichment analysis (GSEA). Single-sample gene set enrichment analysis was employed to estimate immune cells related to this prognostic model. Finally, we constructed a transcriptional factor (TF) network and a competing endogenous RNA regulatory network.

**Results:** Twenty-three differentially expressed DDR genes were detected between TNBC and normal samples. The six-gene prognostic model we developed was shown to be related to OS in TNBC using univariate and LASSO Cox regression analyses. All the six DEGs were identified as significantly up-regulated in the tumor samples compared to the normal samples in qRT-PCR. The GSEA analysis indicated that the genes in the high-risk group were mainly correlated with leukocyte migration, cytokine interaction, oxidative phosphorylation, autoimmune diseases, and coagulation cascade. The mutation data revealed the mutated genes were different. The gene-TF regulatory network showed that Replication Factor C subunit 4 occupied the dominant position.

**Conclusion:** We identified six gene markers related to DDR, which can predict prognosis and serve as an independent biomarker for TNBC patients.

## Introduction

Breast cancer is the most common malignancy in women. The International Agency for Research on Cancer of the World Health Organization reported that the number of new cases of breast cancer reached 2.26 million in 2020, and breast cancer has become the most prevalent malignant tumor in the world (Sung et al., 2021). Breast cancer is a biologically and clinically heterogeneous disease with several recognized tissue and molecular subtypes with different etiologies, risk factors, treatment responses, and prognoses (Allemani et al., 2018; Mavaddat et al., 2019; Pashayan et al., 2020). Triple-negative breast cancer (TNBC) is defined as a type of breast cancer with negative expression of estrogen (ER), progesterone (PR), and human epidermal growth factor receptor-2 (HER2; Bianchini et al., 2016). Compared with other breast cancer subtypes, TNBC is highly aggressive and has a high rate of early recurrence. Patients with TNBC usually relapse within 5 years after surgery, and the overall prognosis is very poor (Emens, 2018). Due to the lack of ER, PR, and HER2 expression in TNBC tumors, few therapies targeting specific molecular targets have been able to significantly improve the prognosis of patients with TNBC disease, and chemotherapy remains the standard of treatment of TNBC (Bianchini et al., 2016; Lee et al., 2020). Although many patients with early-stage TNBC disease are cured by chemotherapy, the overall median survival with the current treatment regimen is 13–18 months among those who develop metastatic disease (André and Zielinski, 2012). Therefore, in order to improve the prognosis and curative effect of TNBC patients, it is urgent to obtain new and effective biomarkers.

DNA damage response (DDR; Cancer Genome Atlas Research Network, 2017. Electronic address and Cancer Genome Atlas Research) pathways are an important mechanism to correct and repair DNA damage, which can inhibit cell aging, apoptosis, and carcinogenesis in time and ensure normal life activities (Roos et al., 2016). DDR consists of eight pathways: (1) mismatch repair (MMR), (2) base excision repair (BER), (3) nucleotide excision repair (NER), (4) homologous recombination repair (HRR), (5) non-homologous end ligation (NHEJ), (6) checkpoint factor (CPF), (7) Fanconi’s anemia (FA), and (8) variable DNA synthesis (TLS). The interaction of these pathways can repair DNA damage accurately and timely, prevent gene distortion, and ensure the integrity of the genome (Scarbrough et al., 2016). Recent studies have shown that increasing DNA damage and reducing the DNA repair ability of cancer cells lead to genome distortion of cancer cells, but that distinguishing these cells from normal cells can improve the effectiveness of cancer treatment (Lawrence et al., 2014). DDR genes can broaden therapy options for cancer patients by cancer-driving effects and significance in clinical and translational medicine (Hu and Guo, 2020). For example, DDR alterations are independently associated with the therapeutic response to PD-1/PD-L1 inhibitors and are positively correlated with a higher tumor mutation burden (Turner et al., 2004; Cerrato et al., 2016). Poly-ADP-ribose polymerase inhibitor therapy had a better effect on cancer patients with BRCA1/2 mutations (Faraoni and Graziani, 2018; Teo et al., 2018). Moreover, many studies have demonstrated that tumors with deleterious DDR mutations are more sensitive to platinum-based therapy (Tutt et al., 2018). Therefore, DDR genes are very important to the prognosis of patients, but there has yet to be a systematic study of DDR genes in TNBC.

In this study, we downloaded the sequencing data and DDR datasets of TNBC patients from The Cancer Genome Atlas (TCGA) and the Gene Expression Omnibus (GEO) databases and performed bioinformatics analysis on them to comprehensively evaluate whether the expression level of DDR-related genes can predict the prognosis of TNBC patients. The aim of our study was to identify new potential prognostic markers for TNBC and establish new prognostic models to assist in the formulation of diagnosis and treatment strategies. In addition, we stratified the risk of TNBC patients by establishing a prognostic model, and then performed more specific treatments on the patients according to the results of the different risk assessments, so as to avoid unnecessary active treatments for the patients.

## Materials and Methods

### Clinical Sample Acquisition

Tumor tissue samples and adjacent normal breast tissue samples from 10 TNBC patients were obtained from the sample library of The First Affiliated Hospital of Zhengzhou University, Henan, China. All the patients had received surgery in The First Affiliated Hospital of Zhengzhou University and had not received any anti-cancer treatment before surgery. Tissue specimens were collected within 30 min after surgery and quickly frozen in liquid nitrogen. Postoperative monitoring and treatment continued in accordance with the relevant consensus guidelines. Tumors were graded according to the WHO grading system. Each patient’s written informed consent to donate the sample to the sample bank was required before any sample was collected. Our research was approved by the Ethics Committee of The First Affiliated Hospital of Zhengzhou University and was conducted in accordance with the principles of Declaration of Helsinki. The age and clinical conditions of each patient, such as immunohistochemistry, are shown in Supplementary Table 1.

### Data Collection and Differential Expression Analysis

The gene expression data and clinical information of TNBC patients were acquired from the TCGA database^1^ and GEO database^2^. We collected 99 normal and 113 TNBC samples from the TCGA database. Differentially expressed genes (DEGs) were selected using the “limma” package (|log_2_ (fold change)| > 1, *P* < 0.05). We obtained six DDR gene sets that encompassed several DDRs pathways, including MMR, BER, CPFs, NER, HRR, FA, and NHEJ, from Molecular Signatures Database (MSigDB)^3^ and 57 duplicate DDRs were removed. Finally, we collected 154 DDR genes and intersected them with DEGs (DE-DDRs).

### Survival Analysis

From a total of 113 TNBC samples, after excluding a TNBC sample lacking survival data, we used 112 TNBC samples to construct a prognostic model. Univariate and LASSO Cox analyses were used to identify prognostic genes that were significantly associated with OS (overall survival). Univariate Cox analysis was used to initially identify the potential prognostic genes (*P* < 0.2; Kang et al., 2013). Next, we used the R package “glmnet” for the LASSO Cox regression analysis to construct the prognostic model for TNBC patients. The risk score was calculated according to the standardized expression level of each gene and its corresponding regression coefficient. The following formula was used: Risk score = (Coefficient_mRNA1_ × Expression_mRNA1_) + (Coefficient_mRNA2_ × Expression_mRNA2_) + ⋯ + (Coefficient_mRNAn_ × Expression_mRNAn_). Setting the median risk score of the TCGA database as the cut-off value, 112 TNBC samples were divided into low- or high-risk groups. The R package “survival” was performed to generate the K-M survival curve, and the R package “survivalROC” was used to generate time-dependent receiver operating characteristic (ROC) curves to evaluate the predictive power of the prognostic model. The GSE58812 dataset, which contained 107 TNBC samples, was used for validation of the above results. Univariate and multivariate Cox regression analyses were performed to analyze the independent prognosis of the six-gene prognostic model. All independent prognostic factors were used to construct a nomogram to predict the survival of TNBC patients at 3 and 5 years. In addition, survival analysis of six prognostic genes was performed separately using the TCGA database.

### RNA Extraction and Quantitative Real-Time Polymerase Chain Reaction

Total RNA was isolated from 10 paired tissues using Nuclezol LS RNA Isolation Reagent (ABP Biosciences Inc) according to the instructions. Then, we quantified the concentration and purity of the RNA solution using an Ultraviolet spectrophotometer (Life Real). Briefly, the extracted RNA was reverse-transcribed to cDNA using the SureScript-First-strand-cDNA-synthesis-kit (GeneCopoeia) prior to quantitative real-time polymerase chain reaction (qRT-PCR). QRT-PCR reaction system consisted of 4 μl of reverse transcription product, 0.5 μl of BlazeTaq^TM^ SYBR^®^ Green qPCR Mix 2.0 (GeneCopoeia), 0.5 μl each of forward and reverse primers, and 3 μl Nuclease-Free Water. PCR was performed in a Mini Amp Thermal Cycller under the following conditions: pre-denaturation at 95°C for 30 s; 40 cycles of denaturation at 95°C for 10 s; 40 cycles of annealing at 60°C for 20 s; 40 cycles of extension for 20 s. The GAPDH protein was served as an internal control. RNA levels were calculated for tumor samples and paired adjacent samples using the 2^–ΔCt^ method. Primer sequences used for qRT-PCR are shown in Supplementary Table 2.

### Gene Set Enrichment Analysis

To better understand the functional pathways of the high- and low-risk groups, we used GSEA to perform Gene Ontology (GO) and the Kyoto Encyclopedia of Genes and Genomes (KEGG) enrichment analyses. GSEA was performed by using clusterProfiler. *P* < 0.05 was considered statistically significant.

### Mutation Analysis

The somatic mutation data of the 112 TNBC samples were obtained from the TCGA database. We used the “maftools” tool to comprehensively analyze mutation status in TNBC. The “somaticInteractions” function in the R package “maftools” was used to perform a Fisher test on the mutated genes in order to obtain the interaction relationships between them.

### Gene Expression in Pan-Cancer

The expression of six prognostic genes in Pan-Cancer was analyzed by TIMER 2.0^4^, which integrates multiple heterogeneous types of data, including gene symbol, name and location.

### Immune Analysis

The enrichment levels of 28 immune signatures in each TNBC sample were quantified by single-sample gene set enrichment analysis (ssGSEA) in the R package “GSVA.” Heat maps and violin plots were drawn to observe the difference in the level of various immune cell infiltration between the high- and low-risk groups. Finally, the correlation between 6 genes and 28 immune signatures was calculated by the Spearman method.

### Immunohistochemistry and Protein Level Validation

The Human Protein Atlas (HPA) provides information on the tissue and cell distributions of 26,000 human proteins (Uhlén et al., 2015, Uhlen et al., 2017; Thul et al., 2017). We used the HPA database to detect the protein expression level of six prognostic genes by immunohistochemistry (IHC) staining, and obtained IHC images from the HPA database.

### Transcription Factors and ceRNA Network Construction

The Network analyst database^5^ is an online visual analysis platform for gene expression analysis and meta-analysis. In this study, the Network analyst database was used to search the transcriptional factors (TFs) related to the hub genes, which refers to the genes used to construct the prognostic model.

The mRNA-miRNA and miRNA-lncRNA interactions were predicted by using the miRanda database^6^. To improve the reliability of the competing endogenous RNA (ceRNA) network, we used a binding score > 500 and minimal folding free energy (MFE) < − 50 for the predicted mRNA-target miRNA interaction. The screening criteria of miRNA-lncRNA were as follows: binding score > 4,000 and MFE < − 400.

### Statistical Analysis

All analyses were performed using the R software. Univariate and LASSO Cox regression analyses were used to assess the relationship between the prognostic model and OS. The Kaplan–Meier (K–M) method was used to assess survival analysis with a log-rank test. The ROC curves were used to detect the sensitivity and specificity of the prognostic model. *P* < 0.05 was considered statistically as significant.

## Results

### Identification of DE-DDRs

**FIGURE 1:**
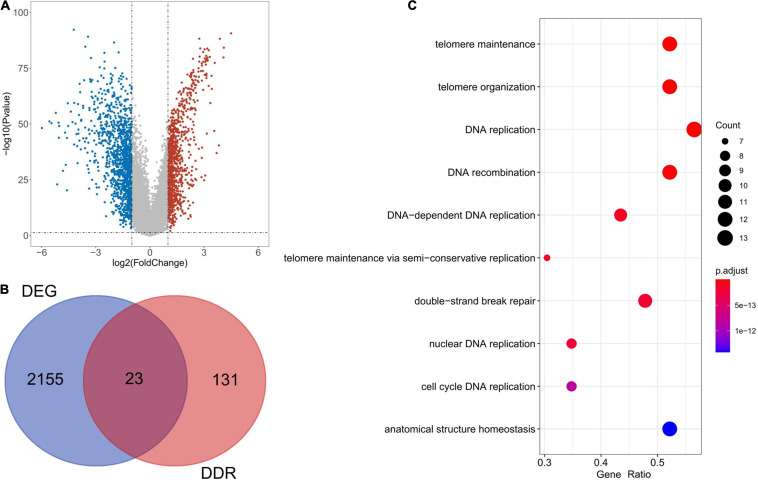
Bioinformatics analysis of the expression of DE-DDRs. **(A)** A Volcano plot illustrating the comparison of differential gene expression in tumors vs. normal tissue. The red dots indicate that the gene expression level is up-regulated (tumor samples relative to normal samples), the blue dots indicate that the gene expression level is down-regulated (tumor samples relative to normal samples), and the gray dots indicate that there is no significant difference between these genes. **(B)** A Venn diagram representation showing the intersection of DDRs and DEGs. **(C)** The results of the GO annotation of DE-DDRs.

Following analysis of the TCGA database using limma, a total of 2,178 DEGs were identified in 113 TNBC and 99 normal samples (| log_2_ (fold change)| > 1, *P* < 0.05). Figure 1A illustrates the 946 up-regulated and 1,232 down-regulated genes using a volcano plot. As shown by the Venn diagrams in Figure 1B, we selected 23 significant DE-DDRs (the intersection of 154 DDRs and 2,178 DEGs) for subsequent analysis. GO function annotation of the DE-DDRs was performed by the R package. These genes were mainly enriched in DNA replication, DNA recombination, and DNA-dependent DNA replication (Figure 1C).

### Construction of Prognostic Model in the TCGA Database

**FIGURE 2:**
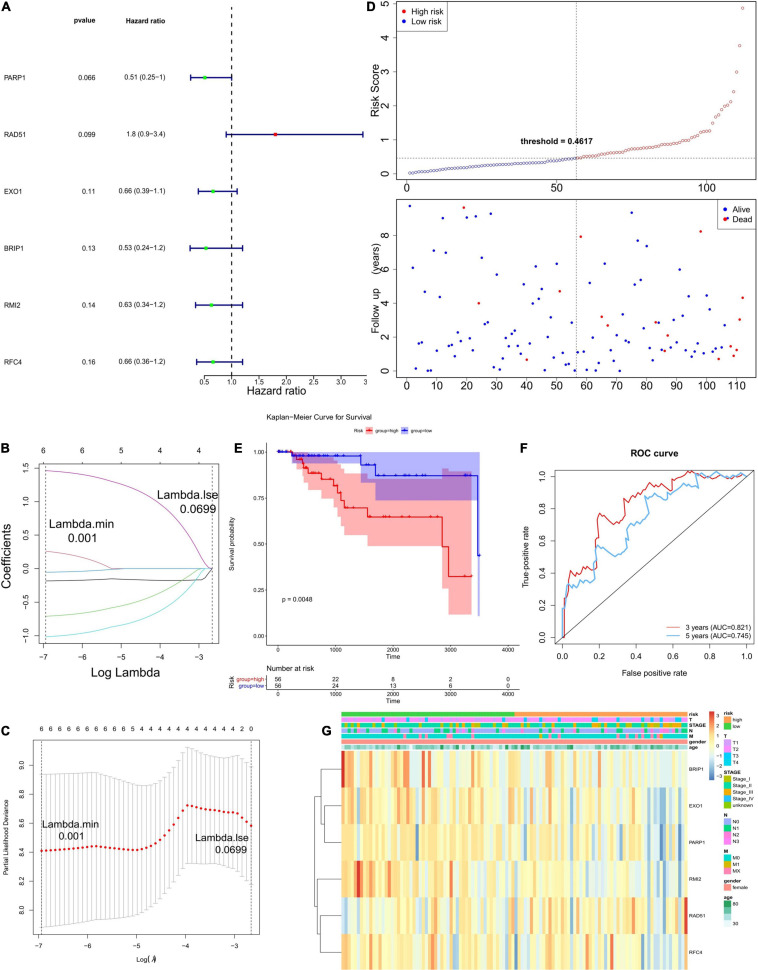
Establishment and evaluation of the prognostic model. **(A)** The forest plots illustrate univariate Cox analysis of the six genes significantly associated with OS. **(B,C)** LASSO coefficient profiles of six genes significantly associated with OS. **(D)** The Risk curve of the risk model. The risk value of patients increases from left to right. According to the median value, the samples were divided into high- and low-risk groups. **(E)** K–M survival curve of the Risk score. In the figure, the ordinate indicates the survival rate, and the abscissa indicates the total survival time. The red curve represents the high-risk group, and the blue curve represents the low-risk group. The difference between high- and low-risk groups was 0.0048, indicating a significant difference. **(F)** The ROC curve used to evaluate the effectiveness of the risk model. **(G)** The top of the heat map shows different clinical characteristics, in which the first line denotes the high-low risk grouping, orange represents the low-risk group samples, and green represents the high-risk group samples. The tree on the left shows the clustering analysis results of different genes from different samples.

In order to establish a prognostic model, univariate Cox regression analysis was performed on 23 genes, of which six genes were significantly associated with the OS of TCGA-TNBC (Figure 2A). The regression coefficients of these six genes were calculated via LASSO COX regression analysis (Figure 2B). The prognostic model achieved the best performance when the six genes were used (Figure 2C). The formula of the model was: risk score = (−0.18330185 × expression level of PARP1) + (0.25938239 × expression level of BRIP1) + (−0.71002582 × expression level of RMI2) + (−0.05379813 × expression level of RFC4) + (−1.01590214 × expression level of EXO1) + (1.46313437 × expression level of RAD51). According to the median risk score, 56 of the 112 TNBC samples were classified into the high-risk group (*n* = 56), and the remaining 56 samples were classified into the low-risk group (*n* = 56; Figure 2D). Survival analysis indicated that the OS was lower in the high-risk group than the low-risk group (*P* < 0.05; Figure 2E). The time-dependent ROC curves were used to evaluate the prediction effect of the risk score, and the AUC was 0.821 at 3 years and 0.745 at 5 years (Figure 2F). The relationships between risk score and clinicopathological features (age, sex, pathological stage, and TNM stage) are shown in Figure 2G.

### Validation of Prognostic Genes Based on Clinical Samples

**FIGURE 3:**
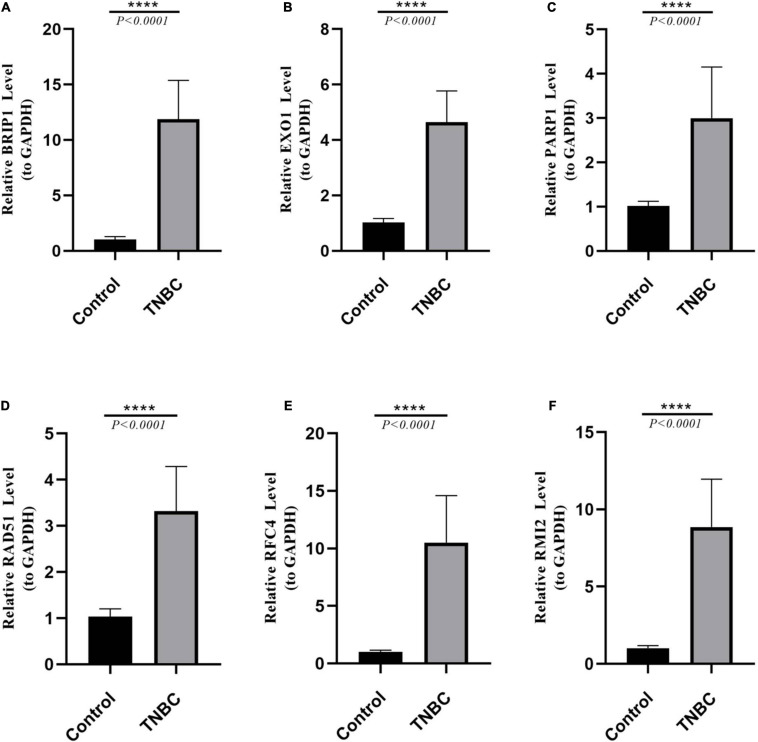
Results of quantitative real-time PCR (qRT-PCR) validation of six differentially expressed genes (DEGs) in TNBC tumor tissues (TNBC) and adjacent normal tissues. The expression of each of these six DEGs was significantly increased in tumor tissues (*P* < 0.0001). Expression levels measured by qRT-PCR analysis of **(A)** *PARP1*. **(B)** *RAD51*. **(C)** *EXO1*. **(D)** *BRIP1*. **(E)** *RMI2*. **(F)** *RFC4*.

Quantitative real-time polymerase chain reaction analysis were performed to assess the expression levels of the six DEGs that constructed our prognostic model. Consistent with the results of the bioinformatics analysis, all DEGs were identified as significantly up-regulated in the tumor samples compared to the normal samples (Figure 3). The results of qRT-PCR analysis were showed in Table 1.

**TABLE 1:** Results of quantitative real-time PCR (qRT-PCR).

	**Control**	**TNBC**	***t***	***P***
PARP1	1.0150.103	2.9971.094	*t* = 5.410 df = 18	<0.0001
RAD51	1.0320.162	3.3200.914	*t* = 7.394 df = 18	<0.0002
EXO1	1.0230.1387	4.6441.065	*t* = 10.11 df = 18	<0.0003
BRIP1	1.0460.235	11.8753.326	*t* = 9.742 df = 18	<0.0004
RMI2	1.0240.153	8.8552.942	*t* = 7.974 df = 18	<0.0005
RFC4	1.0200.126	10.5033.882	*t* = 7.324 df = 18	<0.0006

### Validation of the Six-Gene Prognostic Model in the GEO Database

**FIGURE 4:**
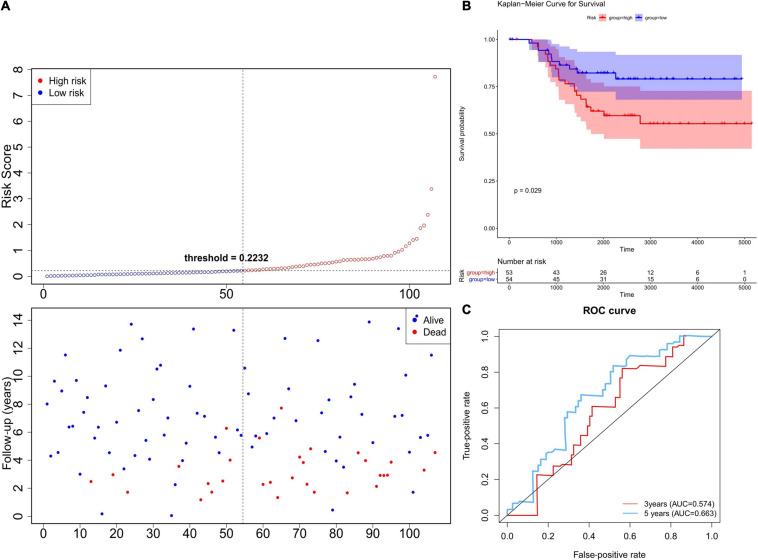
Validation of the six-gene prognostic model using the GEO database. **(A)** The Risk curve of the risk model. The risk value of patients increases from left to right. According to the median value, the samples were divided into high- and low-risk groups. **(B)** K–M survival curve of verification set—Risk score. The ordinate indicates survival rate, and the abscissa indicates total survival time. The red curve represents the high-risk group, and the blue curve represents the low-risk group. The difference between high- and low-risk groups was significant (0.0012). **(C)** Validation Set—ROC Curve to evaluate the effectiveness of the risk model.

In order to verify the robustness of the prognostic model, we applied the model to the GEO cohort for external validation. Patients in the GSE58812 dataset (*n* = 107) were divided into a high-risk group (*n* = 53) and low-risk group (*n* = 54) using the formula obtained from TCGA-TNBC cohort (Figure 4A). Consistent with the TCGA cohort, the survival probability of high-risk patients was significantly lower than that of low-risk patients (Figure 4B). As shown in Figure 4C, the AUC of the ROCs was 0.574 for 3 years and 0.663 for 5 years. Since there were only three patients with data regarding the 1-year follow-up, we did not plot the ROC curve of the 1-year follow-up for the TCGA and GSE58812 datasets. Collectively, these results indicated that the six-gene prognostic model was robust for survival prediction.

### Independent Prognostic Value of the Six-Gene Prognostic Model

**FIGURE 5:**
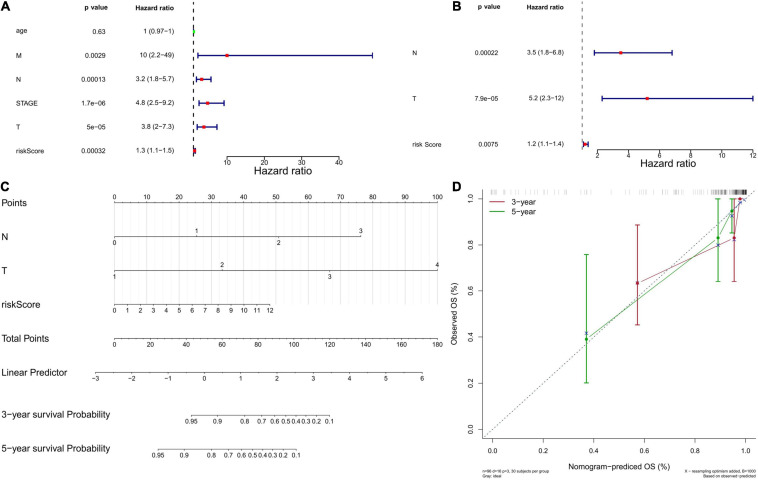
Independent prognostic value of the six-gene prognostic model. **(A)** Independent prognostic factors as determined by the Univariate Cox regression analysis. The left side represents the gene, corresponding *P* value, and HR value. The red square on the right indicates that the HR value is greater than 1, the green square indicates that the HR value is less than 1, and the line segments on both sides of the square are 95% confidence intervals of the HR value. **(B)** Independent prognostic factors as determined by the Multiple Cox regression analysis. **(C)** The nomogram to predict overall survival was created based on three independent prognostic factors. Each factor corresponds to a score, and the sum of the total scores of each factor corresponds to the total score. The 1-year, 3-year, and 5-year survival rate is predicted according to the total score. **(D)** The correction curve based on the above prediction model. The c-index of the model is 0.902371, and the corrected c-index is 0.887527.

Next, we used univariate and multivariate Cox regression analyses to evaluate whether the six-gene prognostic model could serve as an independent predictor for TNBC patients. Univariate Cox regression analysis showed the variables of tumor stage, TNM stage, and risk score were significantly associated with OS (*P* < 0.05; Figure 5A). Multivariate Cox regression analysis indicated that N stage, T stage, and risk score were independent risk factors correlated with OS (*P* < 0.05; Figure 5B). Moreover, a nomogram was constructed to predict the possibility of 3-year and 5-year OS in TNBC patients by integrating the six-gene prognostic model with other clinicopathological characteristics (T and N stage). As shown in Figures 5C,D, the nomogram and calibration curve demonstrated that the six-gene prognostic model was a valuable indicator for prognostic prediction.

### Separate Survival Analysis of the Six DEGs in TNBC

In TCGA data, we analyzed the effects of the six DEGs on the OS of TNBC patients, but none of them had significant effect on the OS (overall survival) of TNBC patients (Supplementary Figure 1). The results were shown in Supplementary Table 3.

### Expression of Six DEGs in 33 Pan-Cancers

Using the TIMER 2.0 database, we analyzed the expression levels of these six genes at the pan-cancer level, and the results were as follows: *BIRP1*, *PARP1*, and *RFC4* were significantly overexpressed in BRCA (Breast cancer), BLCA (Bladder Urothelial Carcinoma) and LIHC (Liver hepatocellular carcinoma) while they were significantly lower expressed in KICH (Kidney Chromophobe). Compared with normal samples, *EXO1*, *RMI2*, and *RAD51* were significantly overexpressed in most cancer types including BRCA, while *RMI2* was significantly lower expressed in PARD (Prostate adenocarcinoma; Supplementary Figure 2).

### Gene Set Enrichment Analyses

**FIGURE 6:**
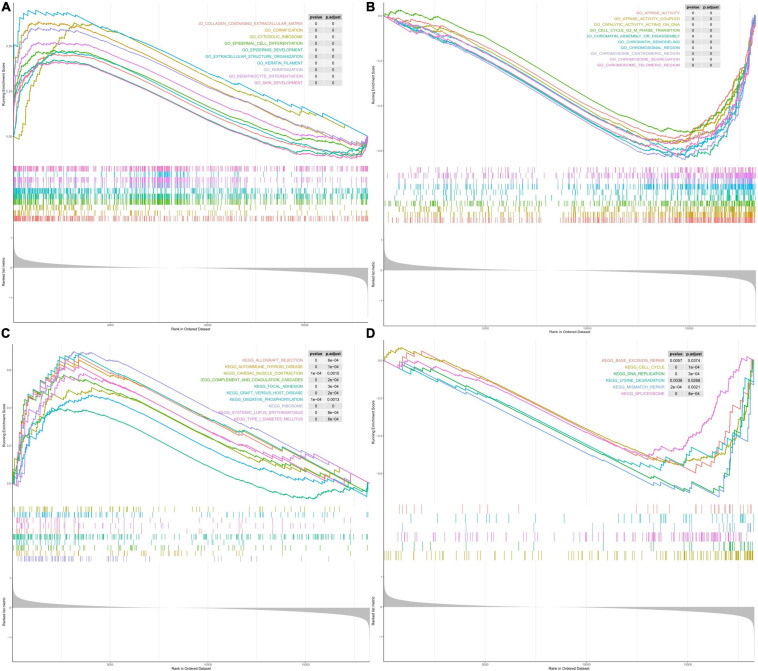
Gene set enrichment analysis (GSEA). **(A)** The top 10 enriched GO pathways in the high-risk group. **(B)** The top 10 enriched GO pathways in the low-risk group. **(C)** The top 10 enriched KEGG pathways in the high-risk group. **(D)** The top 10 enriched KEGG pathways in the low-risk group.

We performed GSEA to identify 672 GO terms and 30 KEGG pathways associated with the high- and low-risk groups in the TCGA cohort (*P* < 0.05). As shown in Figures 6A,B and Supplementary Table 4, the genes in the high-risk group were mainly enriched in leukocyte migration, cytokine interaction with cytokine receptors, oxidative phosphorylation, autoimmune diseases, and coagulation cascade. The genes in the low-risk group were mainly enriched in ATPase activity, chromatin remodeling, DNA replication, methylation, and cell cycle (Figures 6C,D and Supplementary Table 5). In summary, the enrichment analysis revealed potential pathways that could serve as targets in TNBC treatment.

### Clinical Validation of Six Genes in Terms of Protein Expression

**FIGURE 7:**
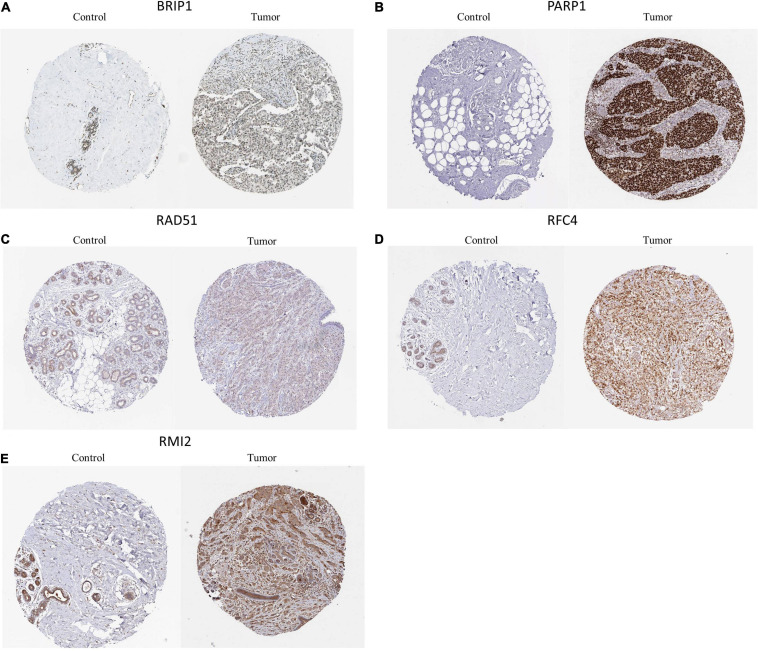
Representative immunohistochemical stains of the six prognostic genes analyzed in the HPA data. **(A)** BRIP1 protein expression in breast cancer and normal control samples. **(B)** PAPR1 protein expression in breast cancer and normal control samples. **(C)** RAD51 protein expression in breast cancer and normal control samples. **(D)** RFC4 protein expression in breast cancer and normal control samples. **(E)** RMI2 protein expression in breast cancer and normal control samples.

The HPA version 9.0^7^ is a public database with millions of immunohistochemical images and was used to compare protein expressions between normal and tumor tissues. Since BRCA data were not classified according to molecular subtypes in HPA, we analyzed the IHC staining of these six DEGs in BRCA to verify the expression level of them. We only found 5 DEGs (*BIRP1*, *PARP1*, *RFC4*, *RMI2*, and *RAD51*) had protein expression data in HPA and the results showed that the expression levels of BIRP1, PARP1, RFC4, RMI2, and RAD51 in BRCA tumor tissues were higher than in normal tissues (Figure 7).

### Landscape of Mutation Profiles in Low-and High-Risk Groups

**FIGURE 8:**
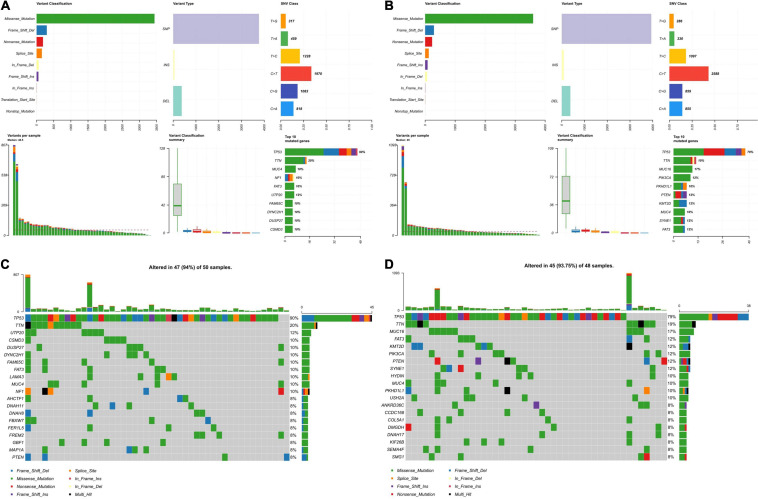
Landscape of mutation profiles in the low-and high-risk groups. **(A)** Overview of mutation types in the high-risk group. **(B)** Overview of mutation types in the low-risk group. **(C)** Waterfall Plot of the top 20 genes with the most mutations in the high-risk group. **(D)** Waterfall Plot of the top 20 genes with the most mutations in the low-risk group.

Since the DDR is closely related to somatic mutations (Tian et al., 2020), we further explored the mutation status of the high- and low-risk groups. After analyzing the mutation data, we found missense mutations accounted for the most mutations in the high- and low-risk groups. The main variant type was single-nucleotide polymorphism, with the most common single nucleotide variants being C > T (Figures 8A,B). Figures 8C,D show the top 20 most frequently mutated genes in the high- and low-risk groups with ranked percentages. The mutation frequency of the two groups was the same (94% vs. 93.75%), while the mutated genes were different. Additionally, the associations across the top 20 mutated genes are shown in Supplementary Figures 3A,B, where green represents co-expression and red represents mutually exclusive relationships.

### Immune Status Between Low- and High-Risk Groups

**FIGURE 9:**
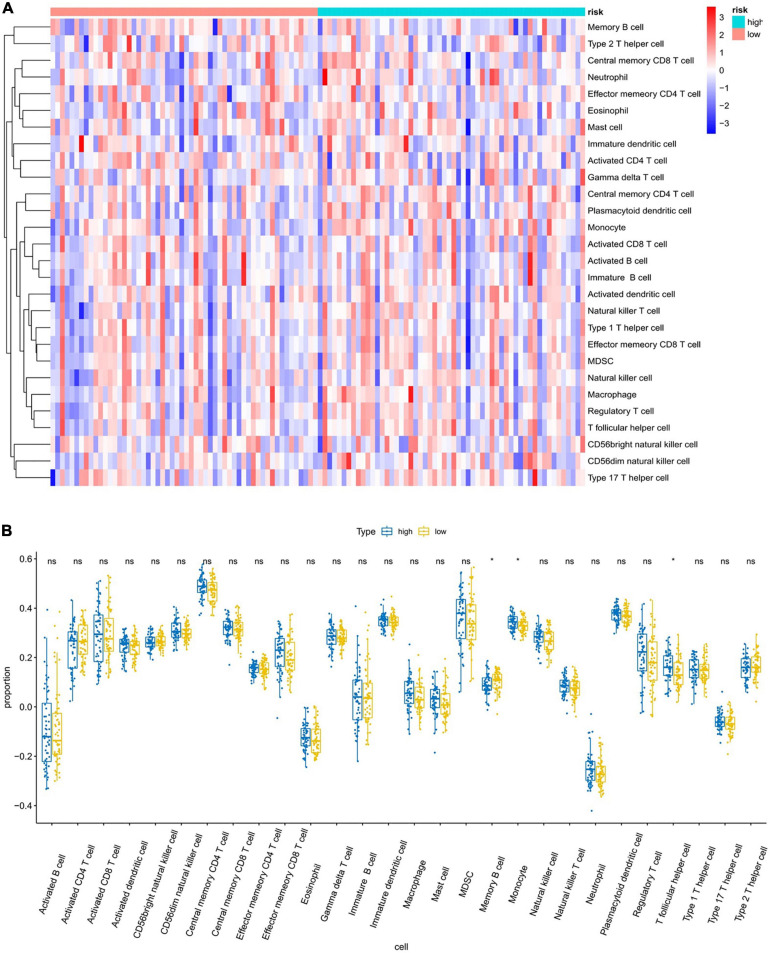
Analysis of immune infiltration patterns in breast cancer samples from TCGA dataset via an ssGSEA score-based method. “ns” represents that there is no significant difference in the infiltration of immune cells between the two samples; “*” represents *P* < 0.05. **(A)** Heatmap of ssGSEA scores (red = positive, blue = negative). **(B)** Boxplot of ssGSEA scores of the 28 representative gene sets.

To further explore the relationship between the six-gene prognostic model and the immune system, ssGSEA was used to evaluate the expression profiles of 28 immune signature genomes in the high- and low-risk groups. The heat map in Figure 9A shows that in the TCGA database, 28 types of immune cells in the low- and high-risk groups showed a certain degree of heterogeneity. The violin plot of the 28 immune cells showed that, as compared with the low-risk group, the content of memory B cells and T follicular helper cells increased, while the content of myeloid-derived suppressor cells (MDSCs) decreased (Figure 9B). We also showed the correlation analysis of six genes and 28 immune cells (Supplementary Figures 4A,B).

### Construction of TF and ceRNA Regulatory Network

**FIGURE 10:**
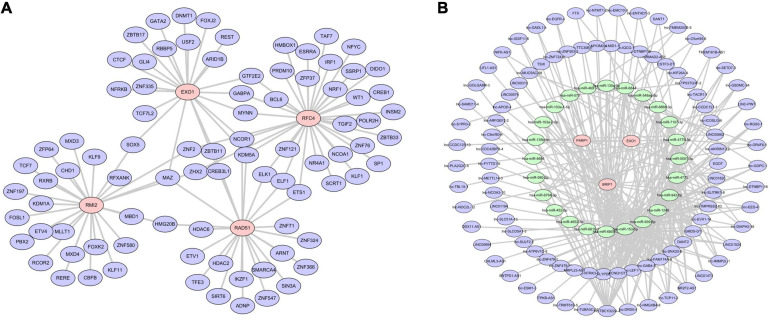
Regulatory network of the risk model genes. **(A)** Transcription factor regulation network diagram. Only four of the six model genes (*RAD51*, *EXO1*, *RMI2*, and *RFC4*) predicted the corresponding transcription factors. In the picture, red is the model gene and blue is TF. **(B)** Transcription factor regulation network diagram. In the picture, red is the model gene, green is miRNA, and blue is lncRNA. Three model genes, 25 miRNAs, and 94 lncRNAs were screened out.

A gene-TF regulatory network was constructed using the Network Analyst database for the six-gene prognostic model we developed in this study. As shown in Figure 10A, we were unable to search for TF related to *BRIP1* and *PARP1*. The constructed transcriptional regulatory network included 105 interaction pairs among 4 genes and 87 TFs, of which *RFC4* regulated most of the TF and occupied the dominant position. Finally, we predicted the target miRNAs of the six-gene prognostic model and lncRNAs that may be related to miRNAs via the miRanda database. A total of 271 lncRNA-miRNA-mRNA pairs were obtained, including 94 lncRNAs, 25 miRNAs, and 3 mRNAs (Figure 10B). It is worth noting that three mRNAs in this ceRNA network including *BRIP1* and *PARP1*, were not found in the Network Analyst database.

## Discussion

DNA is the source of genetic information, and maintaining its integrity is vital to sustaining life. Therefore, cells have evolved specialized DDR mechanisms to maintain the integrity of the genome (Malaquin et al., 2015; Wengner et al., 2020). DDR plays an important role in maintaining homeostasis within the cell (Goldstein and Kastan, 2015). Cancer cells are characterized by genomic instability, which is conducive to the accumulation of driver mutations and the expansion of tumor heterogeneity (Lin et al., 2019; Hu and Guo, 2020). DDR mechanisms can repair mutated genes during the early stage of cancer and hinder the development of tumors (Ali et al., 2017). However, with the development of cancer, DDRs may cause tumor cells to develop resistance to cytotoxic drugs (Brandsma et al., 2017; Carusillo and Mussolino, 2020). The occurrence and development of cancer are often accompanied by the inactivation of one or more DDR pathways; however, cancer cells are therefore more dependent on the remaining DDR pathways than normal cells (Carusillo and Mussolino, 2020). This phenomenon suggests that there are potential weaknesses in tumors that can be targeted by innovative therapies that follow the concept of synthetic lethality. In the concept of synthetic lethality, two pathway defects (independently non-toxic) become fatal when combined (Blackford and Jackson, 2017; Nickoloff et al., 2017; Pilié et al., 2019; Yap et al., 2019). In this study, we screened out six prognostic genes from DDR genes, constructed a risk model based on the bio-analysis of DDR genes, and conducted immune-related analysis based on the model. Our research provides a theoretical basis and reference for the diagnosis and treatment of TNBC.

From the TNBC patient data in the TCGA database, we obtained 2,178 differential genes and intersected them with DDRs genes to obtain 23 DE-DDRs. We then constructed a risk model based on six prognostic genes (*PARP1*, *RAD51*, *EXO1*, *BRIP1*, *RMI2*, and *RFC4*) using univariate analysis and Lasso analysis and determined the effectiveness of the risk model by drawing an ROC curve and a K–M curve. In addition, independent prognostic analysis of the risk model and verification of the model based on the GEO dataset confirmed that the risk model constructed in this study can effectively predict the prognosis of TNBC. In order to investigate the reasons why the model could effectively predict the prognosis of TNBC patients, we conducted enrichment analysis, mutation analysis, and immunocorrelation analysis (immunoinfiltration and immunocorrelation score) on the high- and low-risk groups defined by the model. We found that there were certain differences in immunity between the high- and low-risk groups. Finally, we constructed a TF regulatory network and ceRNA network based on model gene prediction and demonstrated the regulatory function of these key genes.

Poly(ADP-ribose) Polymerase-1 (PARP1) is a member of the PARP family, which has 17 members total and plays a role in various biological functions, including synthetic lethality, DNA repair, apoptosis, necrosis, and histone binding. PARP1, a chromatin-bound nuclear enzyme that is activated by DNA damage, is a validated therapeutic target for cancers and other human diseases (Jain and Patel, 2019; Cao et al., 2020). PARP1 can inhibit the expression of PD-L1 on the surface of TNBC cells by interacting with the nucleic acid-binding domain of nucleophosmin, thus playing a key role in the tumor-related immune escape of TNBC (Qin et al., 2020). In our study, we found that the *PARP1* gene correlated positively with eosinophils.

RAD51 is a strand transferase that aggregates into nucleoprotein filaments on single strands of DNA and promotes the exchange of DNA strands with undamaged homologous chromatin (San Filippo et al., 2008). RAD51 is a component of the cellular DDR, and as such, inhibition of RAD51 sensitizes cancer cells to DNA-damaging drugs (Tsai et al., 2010; Quiros et al., 2011). Studies have found that RAD51 can mediate breast cancer stem cells to develop resistance to PARP inhibitors in TNBC (Liu et al., 2017). In our study, we found a negative correlation between the *RAD51* gene and immune dense cells.

BRIP1, which belongs to the Fanconi anemia (FA) gene family, was first identified via tandem mass spectrometry through its physical interaction with BRCA1 (Rutter et al., 2003). BRIP1 is essential to the stability of the genome, and its normal active expression is necessary for the repair of DNA interstrand cross-links (Moyer et al., 2020). Although pathogenic mutations and a large number of missense mutations in *BRIP1* have been discovered through genetic testing, the impact of these mutations on the molecular function and subsequent role of *BRIP1* in cancer risk is uncertain (Lu et al., 2015; Easton et al., 2016; Weber-Lassalle et al., 2018). Studies have found that *BRIP1* can promote the invasion of breast cancer (BC) cells by regulating the expression of multiple downstream target genes, such as *MGAT5*, *EPCAM*, and *CXCL12*, especially in the triple-negative phenotype MDA-MB-231 cell line (Rizeq et al., 2020). In our study, we found a positive correlation between the *BRIP1* gene and monocytes.

Exonuclease 1 (EXO1) is associated with increased levels of genomic instability in the telomere region, and this widespread genomic instability can promote cancer progression (Maciejowski and de Lange, 2017). EXO1 is a therapeutic target of TNBC that serves an important role in the DDR by inhibiting the activity of PARP (Quist et al., 2019; Li et al., 2021). In our study, we found that the *EXO1* gene has a positive correlation with eosinophils.

RecQ-mediated genome instability protein 2 (RMI2) plays a vital role when the spindle assembly point (Battaglia et al., 2020) is activated during mitosis (Pradhan et al., 2013). RMI2 is widely considered to play a crucial role in DNA damage repair. High expression of RMI2 was confirmed to be associated with the worse prognosis in pancreatic cancer (Xu et al., 2018) and lung cancer (Zhan et al., 2020). RMI2 was also reported to act as a tumor promoter by mediating MYCN/PARP DDR signaling pathway in neuroendocrine prostate cancer (Zhang et al., 2018).

Human Replication Factor C (RFC) is a polyprotein composed of five distinct subunits that are highly conserved through evolution and plays an important role in DNA repair after DNA damage (Kim and Brill, 2001; Krause et al., 2001). Human replication factor C subunit 4 (RFC4) is a member of the RFC family that is often overexpressed in cancer, promoting tumor progression and resulting in worse survival outcomes by regulating tumor cell proliferation and cell cycle. RFC4 has been reported to be overexpressed in a variety of malignancies, including prostate cancer, cervical cancer, colorectal cancer, and head and neck squamous cell carcinoma (Slebos et al., 2006; Narayan et al., 2007; Erdogan et al., 2009; Kang et al., 2009). It can promote tumor progression and lead to worse survival outcomes by regulating cell proliferation and cell cycle arrest in tumors (Yao and O’Donnell, 2012). In our study, we found that the *RFC4* gene had a positive correlation with type 2 T helper cells and a negative correlation with mast cells.

Our results demonstrate that further elucidating the functions of these six DDR-related genes in TNBC may improve our understanding of the biological basis of breast cancer and provide new therapeutic targets. The poor prognosis of TNBC seems to depend on the multi-layered interaction between DNA repair gene mutations, cell proliferation, and the immune response. By including prediction-related biological features, such as immune cells, our six-gene model displayed better predictive value than previously published immune features.

In this study, the correlation between our six gene markers related to the DDR and immune characteristics has been characterized to a certain extent. This model outperforms the prognostic performance of individual clinicopathological prognostic factors and published markers of disease-free survival gene expression, further reinforcing the fact that the immune response is an important component of TNBC. Analyzing the function of the six genetic signatures not only helped us to understand the biological basis of the risk association, but also aided us in making treatment decisions. The main limitations of this study are the retrospective nature of the study and the genes included in the signature were only initially verified by qRT-PCR. More functional validation will be further verified in future experiments and prospective studies.

## Conclusion

We screened and identified six DE-DDRs (*PARP1*, *RAD51*, *EXO1*, *BRIP1*, *RMI2*, and *RFC4*) as prognostic genes through comprehensive bioinformatics analysis and constructed a risk model that can effectively predict the prognosis of TNBC. In addition, we found that the high- and low risk TNBC groups, as defined by the model, exhibited differences in immune-related analysis (immune infiltration, immune-related scores). The above analysis provides a theoretical basis and reference for the research and treatment of TNBC.

## Data Availability Statement

The datasets presented in this study can be found in online repositories. The names of the repository/repositories and accession number(s) can be found in the article/Supplementary Material.

## Ethics Statement

The studies involving human participants were reviewed and approved by Ethics Committee of the First Affiliated Hospital of Zhengzhou University. Written informed consent for participation was not required for this study in accordance with the national legislation and the institutional requirements.

## Author Contributions

NW and YX performed the data analysis and wrote the manuscript. JC, XL, and CZ contributed to the data analysis and manuscript revision. FW and XW contributed to literature search and data extraction. LL and YG proofread the manuscript. NW and LL conceived and designed the study. All authors contributed to the article and approved the submitted version.

## Conflict of Interest

The authors declare that the research was conducted in the absence of any commercial or financial relationships that could be construed as a potential conflict of interest.

## Publisher’s Note

All claims expressed in this article are solely those of the authors and do not necessarily represent those of their affiliated organizations, or those of the publisher, the editors and the reviewers. Any product that may be evaluated in this article, or claim that may be made by its manufacturer, is not guaranteed or endorsed by the publisher.

## Footnotes

1
https://portal.gdc.cancer.gov/cart

2
https://www.ncbi.nlm.nih.gov/gds

3
http://www.gsea-msigdb.org/gsea/msigdb/

4
http://timer.cistrome.org/

5
http://www.networkanalyst.ca/faces/home.xhtml

6
http://www.microrna.org/microrna/home.do

7
http://www.proteinatlas.org

## Abbreviations

AUC: under the ROC curve
BC: breast cancer
BER: base excision repair
ceRNAs: competing endogenous RNAs
CPF: checkpoint factor
DDR: DNA damage response
DEGs: differentially expressed genes
DFS: disease-free survival
EXO1: Exonuclease 1
FA: Fanconi’s anemia
GEO: Gene Expression Omnibus
GO: Gene Ontology
GSEA: gene set enrichment analysis
HER2: human epidermal growth factor receptor-2
HRR: homologous recombination repair
IARC: International Agency for Research on Cancer
KEGG: Kyoto Encyclopedia of Genes and Genomes
MDSCs: myeloid-derived suppressor cells
MFE: minimal folding free energy
MMR: Mismatch repair
MSigDB: Molecular Signatures Database
NER: nucleotide excision repair
NHEJ: non-homologous end ligation
NPM1: nucleic acid-binding domain of nucleophosmin
OS: overall survival
PAPRi: poly-ADP-ribose polymerase inhibitor
PR: progesterone
RFC: Replication Factor C
RMI2: RecQ-mediated genome instability protein 2
ROC: receiver operating characteristic
SAC: spindle assembly point
SNP: single-nucleotide polymorphism
ssGSEA: single-sample gene set enrichment analysis
TCGA: the Cancer Genome Atlas
TF: transcriptional factor
TLS: Translesion Synthesis
TMB: tumor mutation burden
TNBC: triple-negative breast cancer.
