# Supplementary material for: Screening of DNA Damage Repair Genes Involved in the Prognosis of Triple-Negative Breast Cancer Patients Based on Bioinformatics
Source: Front Genet. 2021 Aug 2;12:721873. doi: 10.3389/fgene.2021.721873 (PMC8365772; doi:10.3389/fgene.2021.721873)
Supplement: Supplementary file 6 [file Data_Sheet_1.docx]

Supplementary Material 1

**Supplementary** **Table 1** The age and clinical conditions of each patient, including sampling time, pathological type, tumor staging and immunohistochemical status.

**Supplementary Table 2**  Primer sequences used for qRT-PCR.

**Supplementary Table 3** Survival analysis of 6 prognostic genes performed separately by TCGA database.

**Supplementary Table 4** The key genes were enriched to 672 GO pathways, including ATPase activity, chromatin remodeling, collagen of extracellular matrix, helicase activity, regulation of leukocyte migration and so on.

**Supplementary Table 5** The key genes were enriched into 30 KEGG pathways, including autoimmune thyroid disease, complement and coagulation cascade, systemic lupus erythematosus, antigen processing and presentation, and intestinal immune network for IgA production

**Supplementary Figure 1** Survival analysis of 6 prognostic genes performed separately by TCGA database. Kaplan-Meier survival analysis showed 6 prognostic genes expressions were not significantly associated with overall survival in TNBC patients. Kaplan-Meier survival analysis of **(A)***BRIP1*. **(B)** *EXO1*. **(C)** *PARP1*. **(D)** *RAD51*. **(E)** *RFC4*. **(F)** *RMI2.*

**Supplementary Figure 2**  Expression levels of the 6 prognostic genes in Pan-Cancer from TCGA. The boxplot displays the first quartile, median, and the third quartile of the data. **(A)** *BRIP1*. **(B)** *EXO1*. **(C)** *PARP1*. **(D)** *RAD51*. **(E)** *RFC4*. **(F)** *RMI2.* Abbreviations: ACC: Adrenocortical carcinoma; BLCA: Bladder urothelial carcinoma; BRCA: Breast invasive carcinoma; CESC: Cervical squamous cell carcinoma and endocervical adenocarcinoma; CHOL: Cholangiocarcinoma; COAD: Colon adenocarcinoma; DLBC: Lymphoid Neoplasm Diffuse Large B-cell Lymphoma; ESCA: Esophageal carcinoma; GBM: Glioblastoma multiforme; HNSC: Head and neck squamous cell carcinoma; KICH: Kidney chromophobe; KIRC: Kidney renal clear cell carcinoma; KIRP: Kidney renal papillary cell carcinoma; LAML: Acute Myeloid Leukemia; LGG: Brain Lower Grade Glioma; LIHC: Liver hepatocellular carcinoma; LUAD: Lung adenocarcinoma; LUSC: Lung squamous cell carcinoma; MESO: Mesothelioma; OV: Ovarian serous cystadenocarcinoma; PAAD: Pancreatic adenocarcinoma; PCPG: Pheochromocytoma and Paraganglioma; PRAD: Prostate adenocarcinoma; READ: Rectum adenocarcinoma; SARC: Sarcoma; TGCT: Testicular Germ Cell Tumors; STAD: Stomach adenocarcinoma; THCA: Thyroid carcinoma; THYM: Thymoma; UCEC: Uterine corpus endometrial carcinoma; UCS: Uterine Carcinosarcoma; UVM: Uveal Melanoma.

**Supplementary Figure 3**  Interactions among the top 20 genes with the most mutations. Each square represents an interaction between two genes, and the darker the color, the closer the interaction (green indicates co-expression and red indicates co-exclusion). The dot indicates *P* < 0.05, and the asterisk indicates *P* < 0.001. Among these 20 genes, there are several genes that are co-expressed. **(A)** Interaction of top 20 Genes with Most Mutations in high-risk groups. **(B)** Interactions among the top 20 genes with the most mutations in the low-risk group.

**Supplementary Figure 4** The correlation between risk model genes and immune cells. The abscissa indicates correlation, where a value greater than zero indicates positive correlation and less than zero indicates negative correlation. The ordinate indicates different immune cell types. **(A)** There was a strong positive correlation between the *BRIP1* gene and monocytes. **(B)** The *EXO1* gene had a strong positive correlation with eosinophils. **(C)** The *PARP1* gene had a strong positive correlation with eosinophils. **(D)** There was a strong negative correlation between the *RAD51* gene and immune dense cells. **(E)** The *RFC4* gene had a strong positive correlation with type 2 T helper cells and a strong negative correlation with mast cells. **(F)** The *RMI2* gene had a strong positive correlation with eosinophils and a strong negative correlation with type 17 T helper cells.
