# Supplementary material for: Screening of DNA Damage Repair Genes Involved in the Prognosis of Triple-Negative Breast Cancer Patients Based on Bioinformatics
Source: Front Genet. 2021 Aug 2;12:721873. doi: 10.3389/fgene.2021.721873 (PMC8365772; doi:10.3389/fgene.2021.721873)
Supplement: Supplementary file 7 [file Data_Sheet_2.docx]

Supplementary Material 2

# Abbreviations

AUC: under the ROC curve; BC: breast cancer; BER: base excision repair; ceRNAs: competing endogenous RNAs; CPF: checkpoint factor; DDR: DNA damage response; DEGs: differentially expressed genes; DFS: disease-free survival; EXO1: Exonuclease 1; FA: Fanconi’s anemia; GEO: Gene Expression Omnibus; GO: Gene Ontology; GSEA: gene set enrichment analysis; HER2: human epidermal growth factor receptor-2; HRR: homologous recombination repair; IARC: International Agency for Research on Cancer; KEGG: Kyoto Encyclopedia of Genes and Genomes; MDSCs: myeloid-derived suppressor cells; MFE: minimal folding free energy; MMR: Mismatch repair; MSigDB: Molecular Signatures Database; NER: nucleotide excision repair; NHEJ: non-homologous end ligation; NPM1: nucleic acid-binding domain of nucleophosmin; OS: overall survival; PAPRi: poly-ADP-ribose polymerase inhibitor; PR: progesterone; RFC: Replication Factor C; RMI2: RecQ-mediated genome instability protein 2; ROC: receiver operating characteristic; SAC: spindle assembly point; SNP: single-nucleotide polymorphism; ssGSEA: single-sample gene set enrichment analysis; TCGA: the Cancer Genome Atlas; TF: transcriptional factor; TLS: Translesion Synthesis; TMB: tumor mutation burden; TNBC: triple-negative breast cancer.
