# Supplementary figures and images for: Screening of DNA Damage Repair Genes Involved in the Prognosis of Triple-Negative Breast Cancer Patients Based on Bioinformatics
Source: Front Genet. 2021 Aug 2;12:721873. doi: 10.3389/fgene.2021.721873 (PMC8365772; doi:10.3389/fgene.2021.721873)

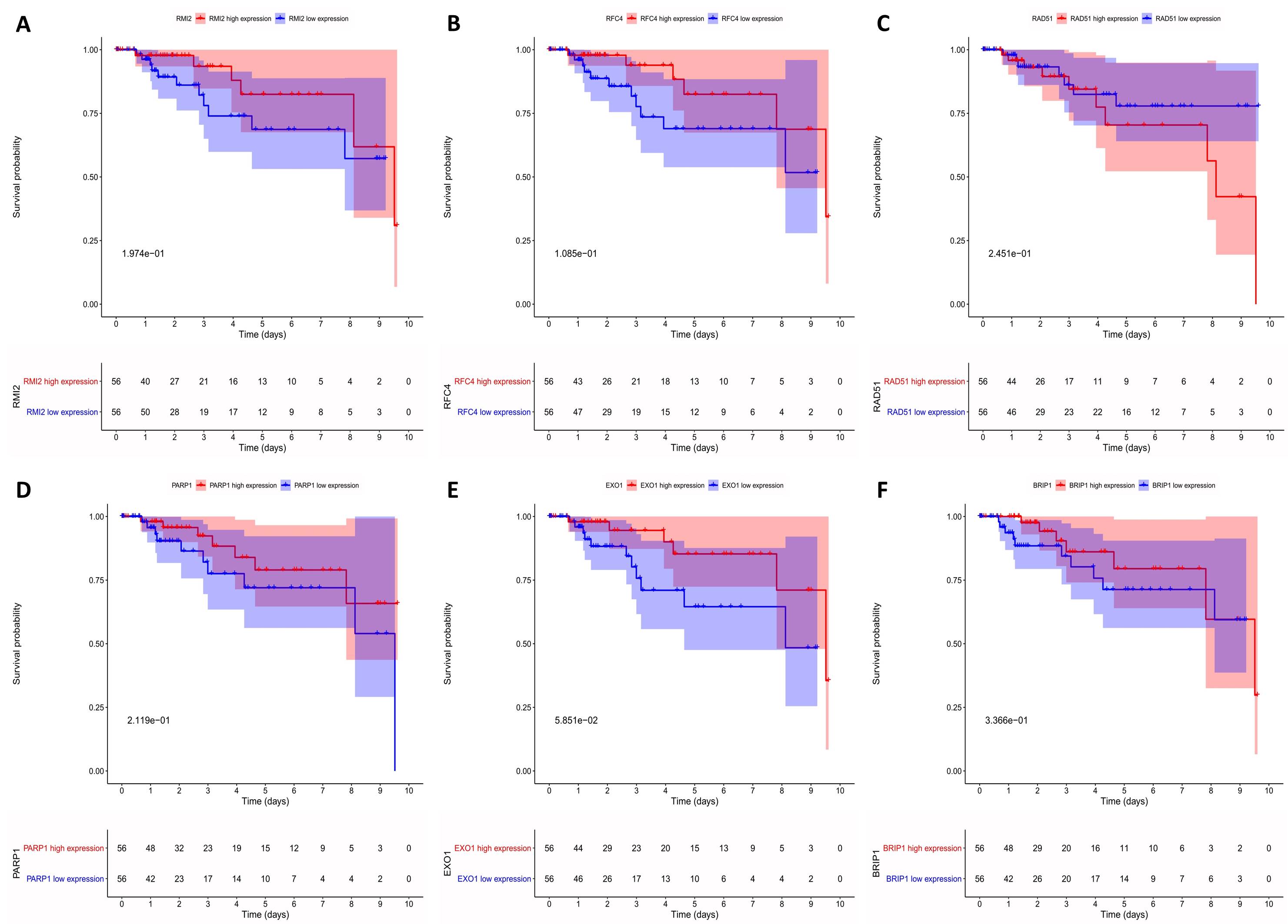

Supplement: Supplementary file 8 [file Image_1.JPEG]

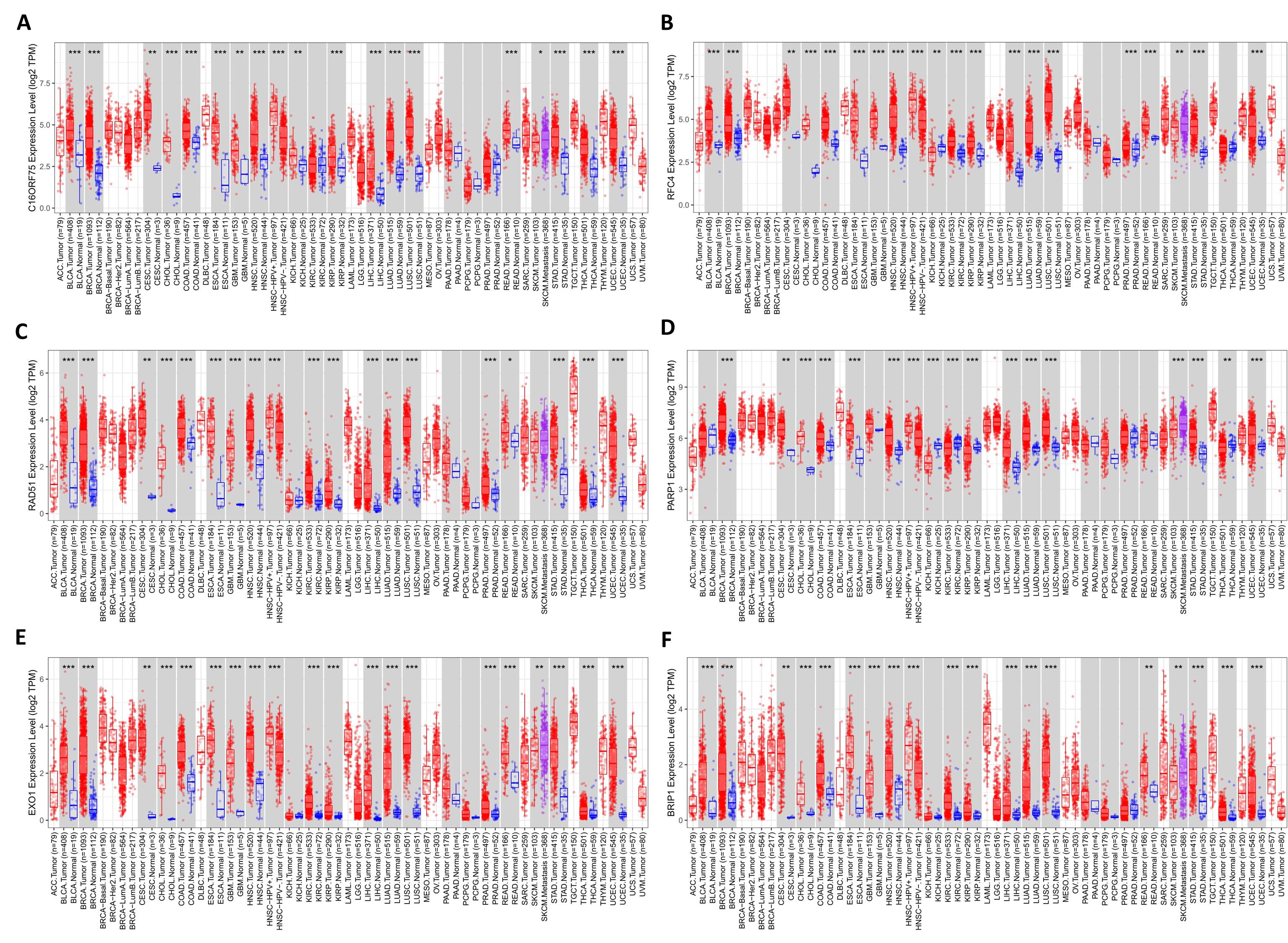

Supplement: Supplementary file 9 [file Image_2.JPEG]

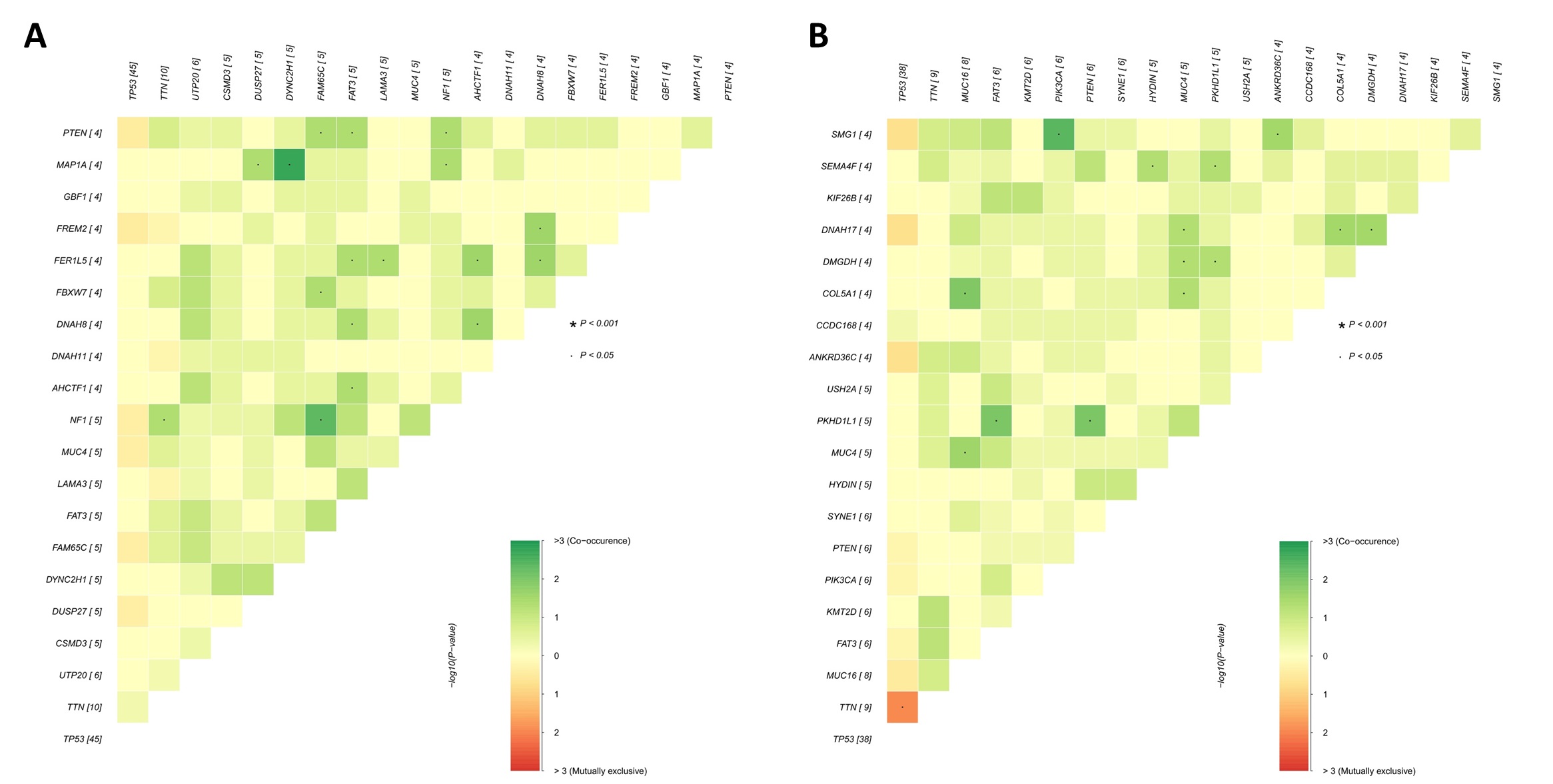

Supplement: Supplementary file 10 [file Image_3.JPEG]

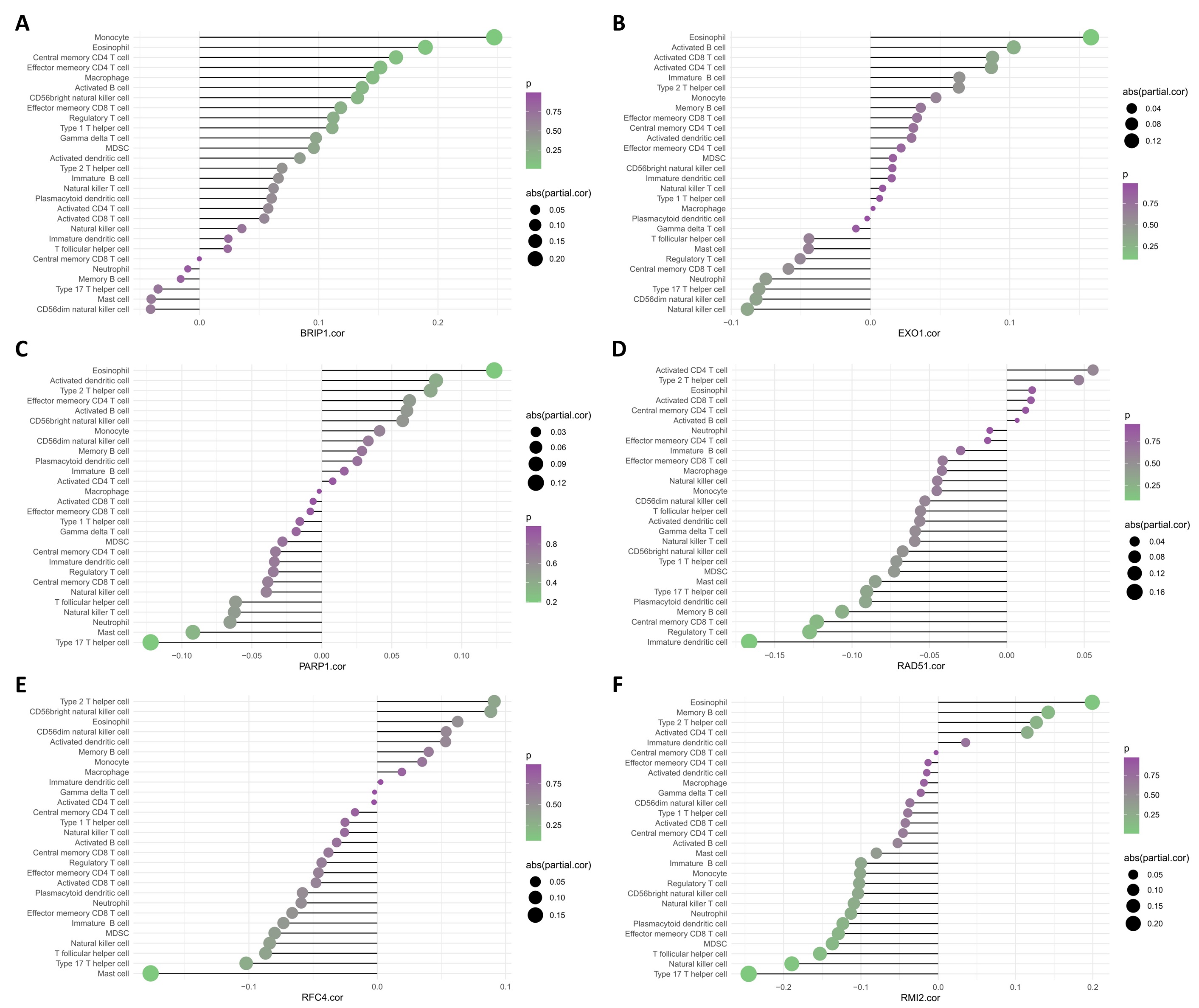

Supplement: Supplementary file 11 [file Image_4.JPEG]
